# NF-κB Pathway as a Potential Target for Treatment of Critical Stage COVID-19 Patients

**DOI:** 10.3389/fimmu.2020.598444

**Published:** 2020-12-10

**Authors:** Ralf Kircheis, Emanuel Haasbach, Daniel Lueftenegger, Willm T. Heyken, Matthias Ocker, Oliver Planz

**Affiliations:** ^1^ Virologik GmbH, Erlangen, Germany; ^2^ Institute of Cell Biology and Immunology, Eberhard Karls University Tuebingen, Tuebingen, Germany; ^3^ Institute for Surgical Research, Philipps University of Marburg, Marburg, Germany

**Keywords:** NF-KappaB, cytokines, chemokines, COVID-19, SARS-CoV-2 (2019-nCoV), proteasome inhibitor, NSAID, cytokine storm

## Abstract

Patients infected with SARS-CoV-2 show a wide spectrum of clinical manifestations ranging from mild febrile illness and cough up to acute respiratory distress syndrome, multiple organ failure, and death. Data from patients with severe clinical manifestations compared to patients with mild symptoms indicate that highly dysregulated exuberant inflammatory responses correlate with severity of disease and lethality. Epithelial-immune cell interactions and elevated cytokine and chemokine levels, i.e. cytokine storm, seem to play a central role in severity and lethality in COVID-19. The present perspective places a central cellular pro-inflammatory signal pathway, NF-κB, in the context of recently published data for COVID-19 and provides a hypothesis for a therapeutic approach aiming at the simultaneous inhibition of whole cascades of pro-inflammatory cytokines and chemokines. The simultaneous inhibition of multiple cytokines/chemokines is expected to have much higher therapeutic potential as compared to single target approaches to prevent cascade (i.e. redundant, triggering, amplifying, and synergistic) effects of multiple induced cytokines and chemokines in critical stage COVID-19 patients.

## Introduction

Coronaviruses—enveloped positive-sense, single-stranded RNA viruses—are broadly distributed in humans and animals. While most human coronavirus (hCoV) infections show mild symptoms, there are highly pathogenic hCoV, including the severe acute respiratory syndrome virus (SARS-CoV) and the Middle East respiratory syndrome coronavirus (MERS-CoV), with 10 and 37% mortality, respectively. The novel coronavirus SARS-CoV-2 with more than 42 mio infected persons and 1.2 mio deaths worldwide (https://coronavirus.jhu.edu/) End of October 2020 has become a global pandemic with enormous medical and socio-economic burden. Patients infected with SARS-CoV-2 show a wide spectrum of clinical manifestations ranging from mild febrile illness and cough up to acute respiratory distress syndrome (ARDS), multiple organ failure, and death, *i.e.* a clinical picture in severe cases that is very similar to that seen in SARS-CoV and MERS-CoV infected patients. While younger individuals show predominantly mild-to-moderate clinical symptoms, elderly individuals frequently exhibit severe clinical manifestations (1–4). Post-mortem analysis showed Diffuse Alveolar Disease with capillary congestion, cell necrosis, interstitial oedema, platelet-fibrin thrombi, and infiltrates of macrophages and lymphocytes (5). Recently, the induction of endotheliitis in various organs (including lungs but also in heart and kidney and intestine) by SARS-CoV-2 infection as a direct consequence of viral involvement and of the host inflammatory response was shown (6, 7).

SARS-CoV-2 binds with its spike (S) protein to the angiotensin-converting enzyme-related carboxypeptidase-2 (ACE-2) receptor on the host cell using the cellular serine protease TMPRSS2 for S protein priming (8). The ACE-2 receptor is widely expressed in pulmonary and cardiovascular tissues, hematopoietic cells, including monocytes and macrophages which may explain the broad range of pulmonary and extra-pulmonary effects of SARS-CoV-2 infection including cardiac, gastrointestinal organs, and kidney affection (6, 8).

## Materials and Methods

### Proteasome Inhibitor

The novel proteasome inhibitor VL-01 (Z-Trp-Trp-Phe-aminohydantoin, MW = 752.82 g/mol) has been described by Leban et al. (9) and was synthesized at Almac (Ireland).

### Confocal Microscopy of Nuclear Translocation of NF-κB

Cells, seeded overnight on cover-slides, were incubated with increasing concentrations of inhibitors, and stimulated with TNFα (2 ng/ml) for 30 min. Cells were fixated with 2% paraformaldehyde, washed, and permeabilized with PBS/Tween®20. Cells were stained using primary antibodies for NF-κB (p65), (rabbit pAb, Santa Cruz, Cat. No.: sc-372, 1:1,000) and fluorescence-labeled secondary antibody [Alexa Fluor® 488 goat anti-rabbit IgG (H+L), Life Technologies Cat. No.: 11008, 1:2,000] for 30 min. Subsequently, the cell nuclei were stained using DAPI (1:40,000, Life Technologies, Cat. No.: D1306) for 30 min. The cover-slides were embedded with Flouramount G (Invitrogen), dried overnight at 4°C, and evaluated by confocal microscope (Leica, LSM3).

### Inhibition of Cytokine Release *In Vivo* in H5N1 Infection Model

The highly pathogenic avian H5N1 influenza A virus strain A/Mallard/Bavaria/1/2006 (H5N1, MB1), obtained from the Bavarian Health and Food Safety Authority, Oberschleissheim, Germany was grown in embryonated chicken eggs.

Six to 8-week-old Balb/c mice from the animal breeding facilities at the Friedrich-Loeffler-Institute, Federal Research Institute for Animal Health, Tuebingen, Germany, were anaesthetized by intraperitoneal injection of 150 µl of a ketamine (1%, Sanofi)-rompun (0,2%, Bayer) solution before treatment. Balb/c mice were intranasal infected with avian H5N1 virus A/mallard/Bavaria/1/2006 (7 × 10exp2 or 100 pfu, i.e. 10-fold MLD50). Mice were i.v. treated with 25 mg/kg VL-01 or solvent (mock) 2 h prior to virus infection. Serum samples for cytokine analysis were collected before and 12, 30, or 72 h after infection. All animal studies were approved by the Institutional Animal Care and Use Committee of Tuebingen.

### Inhibition of Cytokine Release *In Vivo* in LPS Challenge Model

To investigate the effect of VL-01 on LPS induced cytokine response, mice were i.v. treated with 25 mg/kg VL-01 2 h prior to LPS treatment (Lipopolysaccharides from *Escherichia coli* 055:B5, Sigma, Germany, 20 µg/mice). Serum samples for cytokine analysis were collected before (−4 h) and 1.5 and 3 h after LPS treatment.

### Cytokine Analysis

Cytokine analysis was performed using Bio-Plex Protein Arrays from BioRad (Bio-Rad Laboratories, Munich). Bio-Plex-Pro-Mouse Cytokine 6-Plex or 23-Plex were used for cytokine analysis after H5N1 infection or LPS challenge, respectively.

## Results

### Cytokine and Chemokine Storm Is a Hallmark of Acute Respiratory Viral Infections, Such as SARS-CoV-2, SARS-CoV, MERS-CoV, H5N1, and H1N1 (Spanish) Influenza A—Central Role of the NF-κB Pathway

The morbidity and mortality of highly pathogenic hCoV is still incompletely understood. Virus-induced cytopathic effects and viral evasion of the host immune response play a role in disease severity. However, clinical data from patients, in particular those with severe clinical manifestations indicate that highly dysregulated exuberant inflammatory and immune responses correlate with severity of disease and lethality (1, 5–7, 10–12). Significantly elevated cytokine and chemokine levels, *i.e.* cytokine storm, seem to play a central role in severity and lethality in SARS-CoV-2 infections, with elevated plasma levels of IL-1β, IL-7, IL-8, IL-9, IL-10, G-CSF, GM-CSF, IFNγ, IP-10, MCP-1, MIP-1α, MIP-1β, PDGF, TNFα, and VEGF in both ICU (Intensive care unit) patients and non-ICU patients. Significantly higher plasma levels of IL-2, IL-7, IL-10, G-CSF, IP-10, MCP-1, MIP-1α, and TNFα were found in patients with severe pneumonia developing ARDS and requiring ICU admission and oxygen therapy compared to non-ICU patients showing pneumonia without ADRS (1).

Immune profiling of COVID-19 patients revealed distinct immunotypes with therapeutic implications, *i.e*. immunotype 1 characterized by a robust CD4 T cell activation, proliferating effector CD8 T cells was connected to severe disease, immunotype 2 with more traditional effector CD8 T cell subsets, less CD4 T cell activation and memory B cells, showed intermediate clinical outcome, and immunotype 3 with only minimal lymphocyte activation response showed the least clinical symptomatic picture (13). In the same line, asymptomatic SARS-CoV-2 infected individuals exhibited lower levels of a panel of 18 cytokines / chemokines (14).

Detailed insight into the underlying cellular interactions demonstrated by single-cell RNA sequencing analysis showed that COVID-19 severity correlates with the cellular airway epithelium-immune cell interaction. Critical COVID-19 cases—compared to moderate cases—exhibited stronger interaction between epithelial and immune cells, indicated by ligand-receptor expression profiles. Besides expression of pro-inflammatory cytokines, such as IL-1β and TNF-α, the expression of chemokines CCL2, CCL3, CCL20, CXCL1, CXCL3, CXCL10, IL-8 was shown likely to contribute to clinical observation of excessive inflammatory tissue damage, lung injury, and respiratory failure (15).

Regarding the cell types affected, single cell transcriptome and phenotyping studies show that SARS-CoV-2-induced hyper-activation seems to affect a broad spectrum of cells ranging from epithelial cells of the respiratory tract (16–18), lining endothelial cells (6, 7), cells of the innate immune system, including macrophages and mast cells located in the submucosa of the respiratory tract (15, 19), and PBMC (including monocytes, dendritic cells, CD4- and CD8 T-cells) (13, 15, 20).

Notably, also for SARS-CoV and MERS-CoV infected patients, increased levels of pro-inflammatory cytokines in serum, including IL-1β, IL-6, IL-12, IFNγ, TNFα, IL-15, IL-17 and chemokines including CCL2 (MCP-1), CXCL10 (IP-10), CXCL9 (MIG), CCL-5, IL-8 were associated with pulmonary inflammation and extensive lung damage (21–23). Furthermore, both the nucleocapsid protein and the spike protein of SARS-CoV were shown to induce pro-inflammatory cytokines *via* activation of the NF-κB pathway (24, 25). Using comprehensive genomic analyses Smits et al. showed that aged macaques have a stronger host response to virus infection compared to young macaques, with an increase in differential expression of genes associated with inflammation, with NF-κB as central player, whereas expression of type I interferon was reduced indicating a possible negative-feedback cross-talk between the pro-inflammatory NF-κB pathway and IFN-induced antiviral pathways (26).

Interestingly, beside the three highly pathogenic hCoV, also H5N1 and certain H1N1 influenza virus infections with high lethality in humans, showed excessive alveolar immune inflammatory infiltrates and high levels of pro-inflammatory cytokines and chemokines including IP10/CXCL10, IL-6, IL-8, and RANTES in human cell lines, mice, and macaques (27–32) and in humans infected with swine-origin Influenza A virus H1N1 (33).

A differential time-kinetic dependent expression of cytokines and chemokines with acute response cytokines TNFα and IL-1β and chemokines IL-8 and MCP-1 in the early minutes to hours after infection, followed by a more sustained increase in IL-6 was demonstrated by microarray-based transcriptional changes in H5N1 and 1918 H1N1 in comparison to seasonal H1N1 virus infection, with transcriptional changes in the NF-kB pathway central in the cytokine storm (34).

Importantly, recent transcriptome analysis from post-mortem lung tissue of COVID-19 patients and cell culture models infected with COVID-19, Respiratory Syncytial virus and influenza virus identified commonly regulated gene-expression modules of key inflammatory processes for all three acute respiratory viral infections. Key examples were TNF, NF-κB, IL-1, and ALOX5 signaling pathways (17). Several recent reports have demonstrated the NF-κB pathway as *the* central signaling pathway for the SARS-CoV-2 infection-induced pro-inflammatory cytokine/chemokine response (16–18, 35–37). Huang et al. showed in a human *in vitro* model that simulates the initial apical infection of alveolar epithelium with SARS-CoV-2 a rapid transcriptomic change in infected cells, characterized by a shift to an inflammatory phenotype with upregulation of NF-κB signaling and NF-κB target genes by day 1 post-infection, followed by a loss of the mature alveolar program (16). Moreover, SARS-CoV-2 spike protein subunit 1 (CoV2-S1) was shown to induce high levels of NF-κB activations, production of pro-inflammatory cytokines and chemokines (IL-1β, TNFα, IL-6, CCL2), and mild epithelial damage in human bronchial epithelial cells. CoV2-S1-induced NF-κB activation required S1 interaction with human ACE2 receptor and early activation of endoplasmic reticulum (ER) stress, associated unfolded protein response (UPR), and MAP kinase signaling pathways. Notably, a higher activity in NF-κB activation of CoV-2-S1 compared to CoV-S1 was found, probably correlating with the higher binding affinity of CoV-2-S1 to ACE2 receptor (37). Sohn et al. showed that C-C motif (CC) chemokines [CC chemokine ligand (CCL) 2, CCL7, CCL8, CCL24, CCL20, CCL13, and CCL3], C-X-C motif (CXC) chemokines [CXC chemokine ligand (CXCL) 2 and CXCL10], and chemokine receptor subfamilies, as well as IL-1β and its downstream inflammatory signaling molecules (IL1R1, MYD88, IRAK1, TRAF6, NFKBIA, NFKB1, RELA) were dramatically elevated in peripheral blood mononuclear cells (PBMC) from COVID-19 patients compared to healthy controls. Moreover, the expression of the toll-like receptor TLR4 and its related/downstream signaling molecules (CD14, MYD88, IRAK1, TRAF6, TIRAP, TICAM) and most NF-κB signaling pathway genes (NFKBIA, NFKB1, RELA, NFKB2) were significantly upregulated, indicating that TLR4-mediated NF-κB signaling pathway activation is involved in the upregulation of inflammatory responses in patients with COVID-19 infection (38).

Taken together, these multiple reports point to a potential common pathophysiological mechanism of highly dysregulated exuberant inflammatory reactions in response to various acute respiratory RNA virus infections.

### Inhibition of NF-κB Can Inhibit Both Virus-Induced and LPS-Induced Cytokine Storm

**Figure 1:**
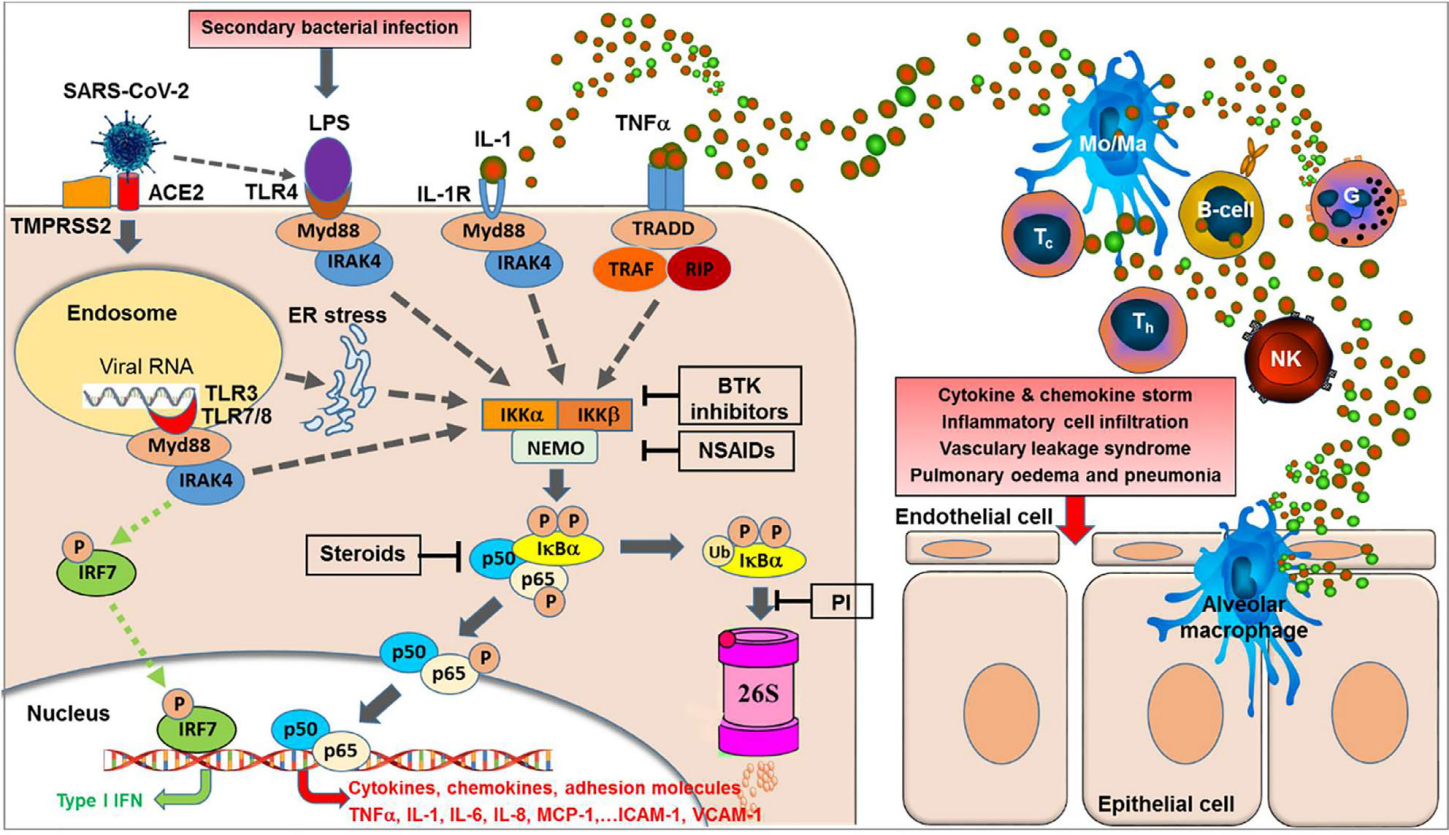
NF-κB activation is central to the acute respiratory RNA virus-induced cytokine storm. Binding of SARS-CoV-2 to its receptor, *i.e.* the angiotensin-converting enzyme 2 (ACE2) and the help of the cellular serine protease TMPRSS2 trigger endocytosis into the host cell. Within the endosomes, RNA from single-stranded RNA virus is known to activate the Toll-like receptors TLR7 and TLR8. Double-stranded RNA intermediates generated during viral replication can be recognized by TLR3. TLR7/8 and/or TLR3 activation can lead to activation of transcription of the interferon-regulator factor (IRF) family and antiviral responses (green dotted lines). However, as a second major effect the activation of the TLRs can trigger—*via* various intermediates—the activation of IKK (IκB kinases) (gray dotted lines) resulting in phosphorylation of the cytoplasmic inhibitor factor IκBα triggering its ubiquitination followed by degradation by the 26S proteasome, thereby NF-κB (a heterodimer complex consisting of protein subunits p50 and p65) is released from IκBα and can now enter the nucleus and initiate transcription of various genes coding for pro-inflammatory proteins such as cytokines, chemokines, adhesion molecules, and growth factors. Importantly, this final sequence of NF-κB activation is shared with a multiple range of cytokine receptor- and Toll-like receptor mediated signal cascades, including binding of TNFα or IL-1 to their receptors or binding of LPS (e.g. from secondary bacterial infections) to TLR4. Furthermore, SARS-CoV-2 was reported to induce TLR4-mediated NF-κB activation as well as ER stress-induced NF-κB activation. Excessive NF-κB activation triggers the gene expression for a broad range of pro-inflammatory cytokines and chemokines, adhesion molecules, and acute phase proteins, resulting in inflammatory cell activation and infiltration, vascular leakage syndrome, finally leading to pulmonary edema and pneumonia. In contrast, interferon-response factor (IRF)-related antiviral responses are largely independent on NF-κB translocation. BTK inhibitors, Bruton Tyrosine Kinase inhibitors; PI, proteasome inhibitors; NSAIDs, nonsteroidal anti-inflammatory drugs; TNFα, tumor necrosis factor-alpha; IL-1, interleukin-1; MCP-1, macrophage chemotactic protein-1; ICAM-1, intercellular adhesion molecule-1; VCAM-1, vascular cell adhesion molecule-1; IRF7, interferon regulatory factor-7; Type I IFN, interferon type I; Myd88, myeloid differentiation primary response 88 protein (adaptor molecule); NEMO, NF-κB essential modulator; IKK, IκB kinase; TRAF, TNF receptor-associated factor; RIP, receptor-interacting protein; TRADD, tumor necrosis factor receptor type-1 associated DEATH domain protein; IRAK4, interleukin-1 receptor-associated kinase 4; ER, endoplasmatic reticulum; LPS, lipopolysaccharide.

We have previously shown that elevated cytokine release of IL-α/β, IL-6, MIP-1β, RANTES, and TNF-α induced by highly pathogenic avian H5N1 influenza A virus was significantly reduced by application of the proteasome inhibitor VL-01 *in vivo* (39). The underlying mechanism of this inhibitory effect of proteasome inhibitors is supposed to be mediated largely by the inhibition of one of the most prominent cellular transcription pathways, NF-κB. The inhibition of the nuclear translocation of the transcription factor NF-κB by proteasome inhibitors has been described (40–42). This is mediated *via* the inhibition of the proteasomal degradation of the cytosolic inhibitor IκBα, this way keeping NF-κB sequestered by IκBα in the cytosol and thereby inhibiting the otherwise induced translocation of NF-κB to the nucleus where it would initiate the transcription of multiple pro-inflammatory proteins, such as cytokines, chemokines, adhesion molecules, and growth factors (**see** **Figure 1**). Activation of the NF-κB pathway has been described for very different signal-receptor bindings, including binding of LPS to Toll-like receptor TLR4, binding of cytokines like IL-1 and TNFα to their respective receptors, or recognition of RNA viruses by Toll-like receptors, TLR7/8 for single stranded RNA or TLR3 for double stranded RNA. TLR7/8 and TLR3 are inserted in membranes in such way that the RNA-recognition domains face towards the extracellular space or into the endosomal lumen (42, 43). For SARS-CoV-2 uptake into the endosomal compartment after binding to the ACE2 receptor (8) has been described and the activation of the endosomal TLR7/8 sensitive to single-stranded RNA is assumed (43). Furthermore, the activation of additional Toll-like receptors, such as TLR2, 3, and 4 has been postulated (44). The activation of TLR3 can be expected by double-stranded RNA intermediates generated during SARS-CoV-2 replication (43). Furthermore, also the activation of TLR4 (at the outer membrane) by SARS-CoV-2 has been indicated by a recent study (38). TLR4 also can be activated by LPD derived from bacterial co-infection or secondary bacterial infections, which have been found in up to 14% of SARS-CoV-2 infected patients (45). Furthermore, a recent study has demonstrated that SARS-Cov-2 spike protein subunit 1 induces high levels of NF-κB activation, production of pro-inflammatory cytokines in human bronchial epithelial cells *via* early activation of endoplasmatic reticulum (ER) stress, and associated unfolded protein response (UPR), and MAP kinase signaling pathways (37).

Importantly, all these different signaling pathways join into a common downstream signaling sequence characterized by phosphorylation of the cytosolic inhibitor IκBα which triggers its ubiquitination and proteasomal degradation resulting in release and translocation of NF-κB into the nucleus (42). This sequence of events in the signal transduction pathways suggests that interfering at these late stages (*i.e.* phosphorylation, ubiquitination, and/or proteasomal degradation of IκBα) of the pathway will inhibit NF-κB activation, irrespectively of the initial triggering signal (see **Figure 1**).

**Figure 2:**
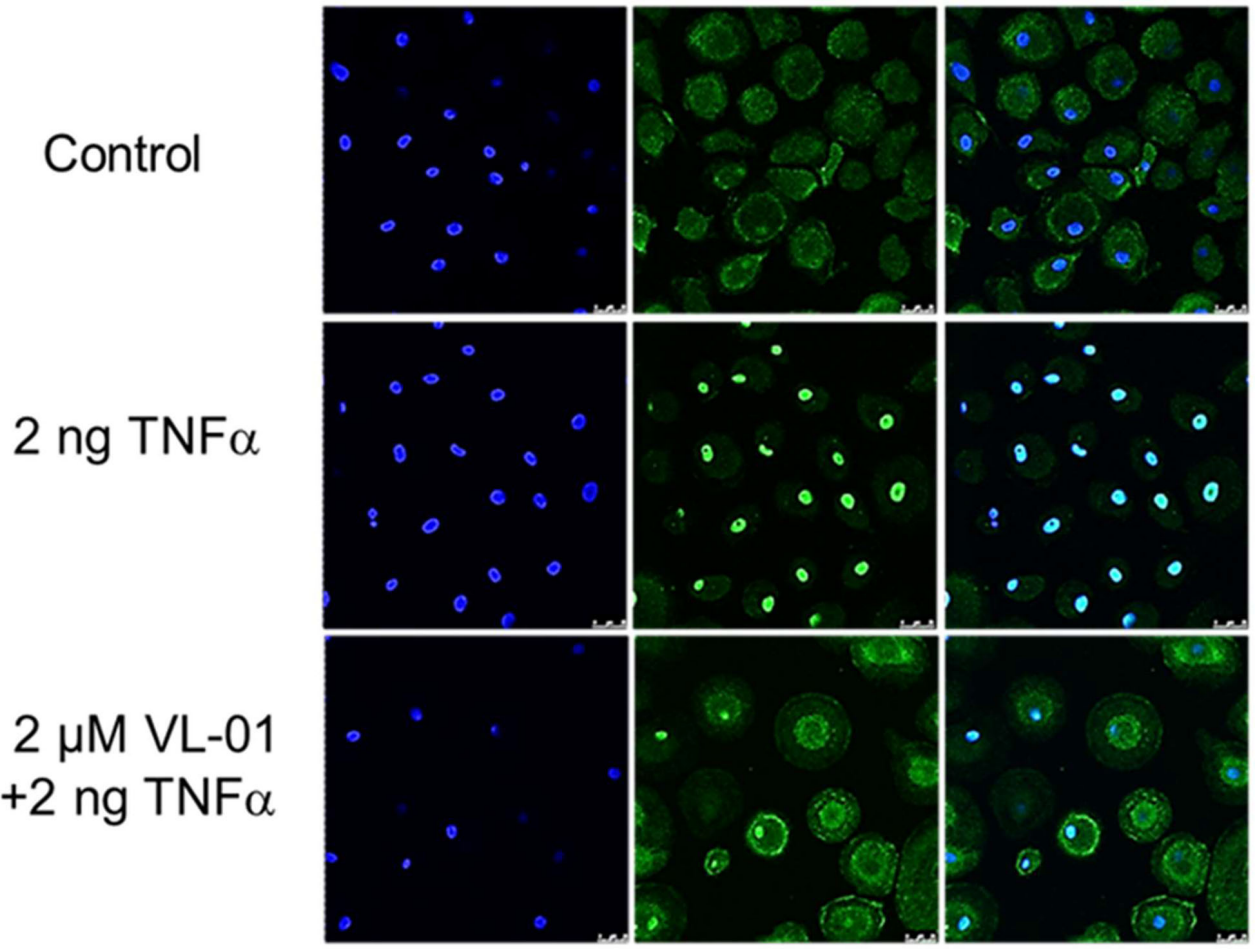
Inhibition of TNFα induced nuclear translocation of NF-κB by the proteasome inhibitor VL-01. Human monocyte-derived macrophages were seeded on cover-slides, incubated overnight, and incubated in the presence or absence of the proteasome inhibitor VL-01 and stimulated with TNFα (2 ng/ml) for 30 min. Immunofluorescence staining of NF-κB was done using a FITC (green)-labeled p65 specific antibody (middle panel), and cell nuclei were counterstained with DAPI (blue, left panel) and evaluated by confocal microscope (Leica, LSM3). Staining of NF-κB (FITC) and nuclei (DAPI) superimposed are shown in the right panel.

We could demonstrate the inhibitory effect of proteasome inhibitors on nuclear translocation NF-κB in various cell types such as human macrophages after stimulation with TNFα *in vitro*. Without stimulation of the NF-κB pathway, p65/p50 (p65 FITC stained) is sequestered in the cytosol by its inhibitor IκB. Following stimulation by TNFα, NF-κB translocates to the nucleus (shown by coinciding p65 staining and nucleus staining by DAPI). NF-κB nuclear translocation after TNFα stimulation was inhibited by application of the proteasome inhibitor VL-01 showing p65 staining in the cytosol and only few cells with p65 positive nucleus (**Figure 2**).

**Figure 3:**
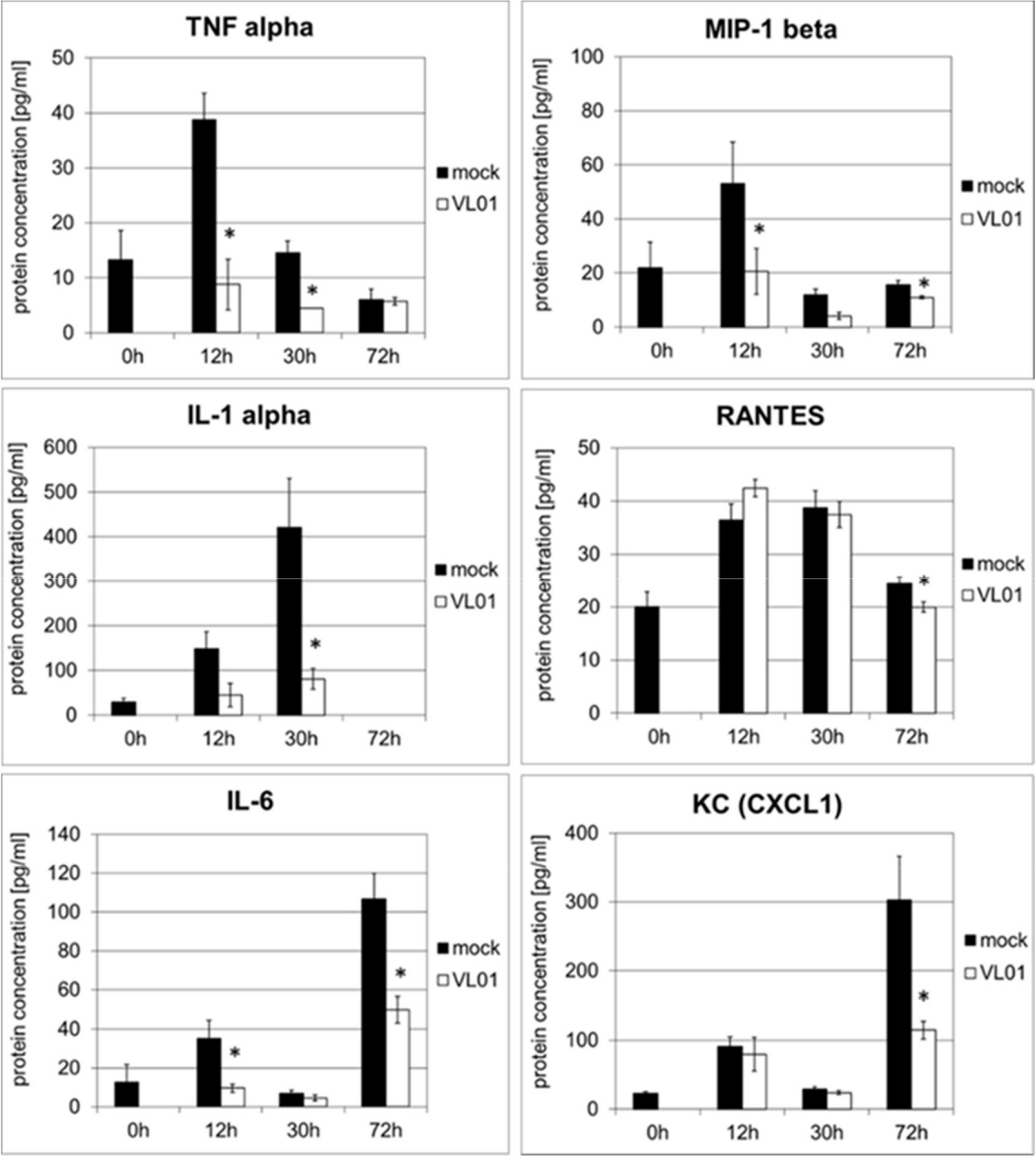
Inhibition of cytokine release in BALB/c mice infected with H5N1 by treatment with the proteasome inhibitor VL-01. Balb/c mice (n = 4) were intranasal infected with avian H5N1 virus A/mallard/Bavaria/1/2006 (7 × 10^2^ pfu, i.e. 10-fold MLD50). Mice were i.v. treated with 25 mg/kg VL-01 or solvent (mock) 2 h prior to virus infection. Cytokine levels in blood were determined before (0 h) and 12, 30, or 72 h after infection using the Bio-Plex Pro Mouse Cytokine 6-Plex Panel (Biorad). *p < 0.05.

The effect of VL-01 on the pro-inflammatory cytokine and chemokine response *in vivo* was demonstrated in a H5N1 influenza virus challenge mouse model. A strong cytokine and chemokine response was induced in Balb/c mice intranasally infected with avian H5N1 virus A/mallard/Bavaria/1/2006 (7 × 10^2^ pfu, i.e. 10-fold MLD_50_). Mice were treated i.v. either with 25 mg/kg VL-01 or solvent (mock) 2 h prior to virus infection. Serum samples for cytokine analysis were collected at different time points after infection. While some cytokines/chemokines such as TNFα and MIP-1β peaked very early after H5N1 infection (12 h), others, *i.e.* IL-1α and RANTES peaked somewhat later (at 30 h), followed by KC (neutrophil-activating protein-3) and IL-6, reaching their peak at 72 h after infection (**Figure 3**). Treatment with proteasome inhibitor significantly inhibited the release of IL-1, IL-6, TNFα, MIP-1β (*i.e.* CCL4), and KC (*i.e*. CXCL1) at their peak time-points in Balb/c mice after infection with the highly pathogenic avian H5N1 influenza A virus (**Figure 3**). Importantly, proteasome inhibition significantly decreased the release for *all*, *early and late* cytokines and chemokines, and resulted in significantly increased survival of mice after infection with the highly pathogenic avian H5N1 influenza A virus (39).

**Figure 4:**
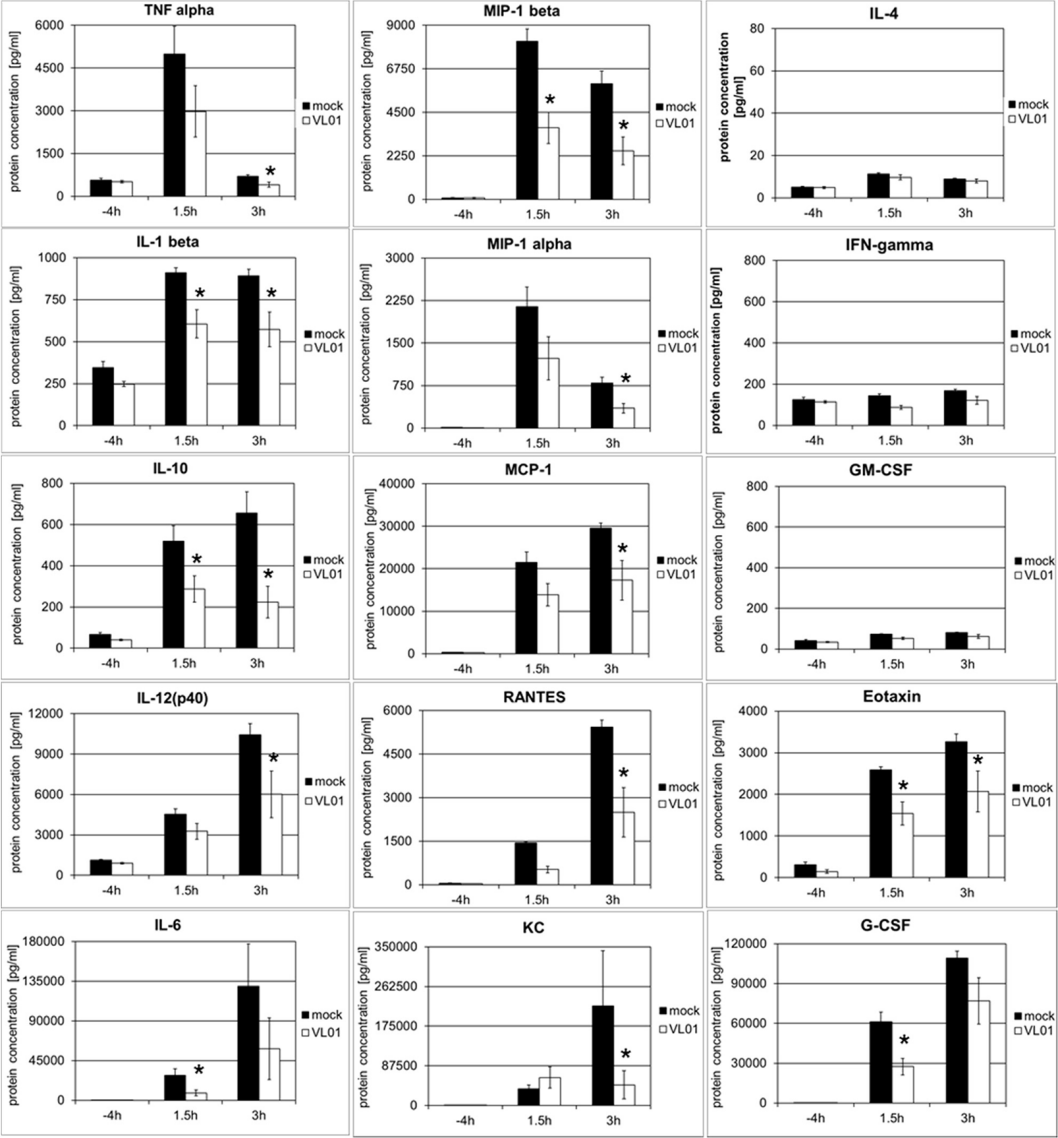
Inhibition of LPS-induced cytokine release in BALB/c mice by the proteasome inhibitor VL-01. Balb/c mice (n = 7) were injected i.v. with VL-01 (25 mg/kg, in 200 µl i.v.) 2 h prior LPS stimulation (20 µg/mouse, 200 ml i.p.). Cytokine levels were determined for the time points before (−4 h), and 1.5 or 3 h after LPS injection, using the Bio-Plex Pro Mouse Cytokine 23-Plex Panel (Biorad). *p < 0.05.

In order to investigate whether the inhibition of cytokine and chemokine release by inhibition of the nuclear translocation of NF-κB is a general mechanism, an acute lung injury (ALI) mouse model with LPS challenge was used. This model provides a rapid and strong systemic induction of pro-inflammatory cytokines and chemokines. Balb/c mice were treated i.v. with 25 mg/kg VL-01, followed by i.p. application of 20 µg LPS. Serum samples for cytokine analysis were collected before (−4 h) LPS treatment (control) and after LPS treatment (1.5 and 3 h). Again distinct release patterns were found for different cytokines/chemokines, with TNFα, IL-1β, MIP-1α (*i.e.* CCL3), and MIP-1β (*i.e.* CCL4) peaking already 1.5 h after LPS challenge, followed by MCP-1 (*i.e.* CCL2), Eotaxin, and G-CSF, further followed by cytokines with more delayed expression, such as IL-6, RANTES (*i.e.* CCL5), IL-12p40, and KC (*i.e.* CXCL1) peaking 3 h after LPS stimulus (**Figure 4**). In contrast, only a minimal or no increase was found for IL-4, IFNγ, and GM-CSF following LPS challenge. The panel of induced cytokines and chemokines in this acute lung injury model is rather similar to the panel of cytokines reported for COVID-19 patients, with cytokines/chemokines such as TNFα, IL-1, IL-6, IL-10, G-CSF, MIP-1α, MIP-1β , and MCP-1 correlating with critical stage in COVID-19 patients (1). Importantly, treatment of mice with proteasome inhibitor significantly reduced the release of the whole panel of the induced pro-inflammatory cytokines (Th1 profile) and chemokines. Taken together, these data generated in different models demonstrate the principal potency of proteasome inhibitors to interfere with the pro-inflammatory effects, by inhibiting the translocation of NF-κB to the nucleus.

As a second line of evidence for the potential role of the NF-κB pathway in acute respiratory viral infection DeDiego et al. have demonstrated, that the inhibition of NF-κB-mediated inflammation in SARS-CoV infected mice significantly decreased the expression of pro-inflammatory cytokines including TNFα, IL-6, and chemokines including CCL2, CCL5, CXCL1, CXCL2, CXCL10, correlating with increased survival. In their study four different NF-κB inhibitors, with different mechanism of inhibition, i.e. CAPE, resveratrol, Bay11-7082, and parthenolide, were used. All four inhibitors were shown to inhibit NF-κB activity, and to decrease the expression levels of pro-inflammatory cytokines and chemokines, without affecting viral titers or cell viability (46).

Moreover, Acetylsalicylic acid (ASA) and other salicylates—in contrast to pure (COX) cyclooxygenase inhibitors, such as indomethacin—are well-known inhibitors of NF-κB activation by acting as specific inhibitors of IKK2 (*i.e.* IKKβ)—a kinase essential for phosphorylating IκB (47). Furthermore, D,L-lysine-acetylsalicylate glycine (LASAG) a water-soluble salt of ASA (licensed as Aspirin i.v.®) was shown to decrease activation of promoter constructs of NF-κB-dependent genes for IL-6 and IL-8 and to improve the time to alleviation of influenza symptoms in hospitalized patients in a phase II clinical trial (48). The well-known analgesic, antipyretic, anti-thrombotic, anti-inflammatory, and antiviral effects of ASA have led to initiation at least eight clinical studies investigating the effects of ASA in COVID-19 according to clinicaltrials.gov (49). A recently published study has demonstrated that indeed Aspirin use is associated with decreased mechanical ventilation, ICU admission, and In-hospital mortality in hospitalized COVID-19 patients (50).

Thirdly, the concept of a central role of NF-κB pathway in critical stage SARS-CoC-2 infected patients is supported by two recently published studies showing pronounced clinical effect in critical COVID-19 patients by Bruton tyrosine kinase (BTK) inhibitors, correlating with significantly decrease in inflammatory parameters (C-reactive protein and IL-6), normalized lymphopenia, and improved oxygenation (51, 52). Bruton tyrosine kinase is known to be involved in TLR7/8-induced TNFα transcription *via* NF-κB recruitment at the stage of phosphorylation of p65 (53).

Finally, support for the role of NF-κB pathway in critical stage COVID-19 patients is provided by recent results from the RECOVERY trial. Dexamethasone was found to significantly reduce death in patients with severe respiratory complications of COVID-19 requiring ventilation by up to one third (54). Dexamethasone—a broadly used glucocorticoid anti-inflammatory drug—is assumed to mediate its anti-inflammatory activity at least partially *via* downregulation of the NF-κB activity (55), probably by suppression of NF-κB expression (56) and/or increased expression of IκB in the cytoplasm (57).

All these data collectively strongly indicate that inhibition of the NF-κB signal pathway may be a promising target to control SARS-CoV-2 induced excessive immune activation associated with systemic cytokine and chemokine release, capillary leakage, and multi-organ tissue damage (**Figure 1**).

## Discussion

Various studies as referenced in the present review have shown that highly stimulated epithelial-immune cell interactions leading to highly dysregulated exuberant inflammatory responses with significantly (topically and systemically) elevated cytokine and chemokine release, play a central role in severity and lethality in various acute respiratory viral infections, including Influenza A H5N1, highly pathogenic H1N1, SARS-CoV, MERS-CoV, and SARS-CoV-2.

Even the higher COVID-19 mortality rate observed in male compared to female patients (58) may correlate with sex differences in the immune responses between male and female, with higher plasma levels cytokines and chemokines including IL-6, IL-8, IL-18, CCL5 found in male patients. In contrast, female patients showed higher IFNα2 levels and a higher T cell activation compared to male patients (59, 60). The reason for these differences may be speculated to be associated with the location of responsive genes on the X chromosomes and accordingly different expression in female and male (61).

Furthermore, while initial reports indicated that children typically have mild or no COVID-19 symptoms and lower rates of hospitalization and death than adults, there is a accumulating number of reports on the occurrence of Multisystem inflammatory syndrome in children (MIS-C) as a newly described condition associated with SARS-CoV-2 exposure that is reminiscent of both Kawasaki disease and toxic shock syndrome (62, 63). A recent study on peripheral immunophenotypes in children with multisystem inflammatory syndrome associated with SARS-CoV-2 infection showed high levels of IL-1β, IL-6, IL-8, IL-10, IL-17, IFN-γ together with high CD64 expression on neutrophils and monocytes, and high HLA-DR expression on γδ and CD4+CCR7+ T cells in the acute phase of MIS-C indicating high immune activation and cytokine release syndrome (64). For treatment of MIS-C blocking of pro-inflammatory cytokines and the use of anti-inflammatory cytokines IL-37 and IL-38 has been suggested as a potential therapeutic tool (65).

A variety of immunomodulatory approaches has been proposed and are being tested to inhibit various cytokines prominently elevated during COVID-19 infection, including monoclonal antibodies against the IL-6 receptor (66–69) or IL-1 receptor antagonist (70, 71). Whereas some clinical efficacy in COVID-19 patients has been recorded also several notable caveats and limitations to the efficacy of single-cytokine targeting approaches have been seen and have led to the question which cytokine to target in a raging storm (72, 73).

The question which particular cytokine / chemokine to target has been shown to be most difficult due to the cascade nature of the induced cytokine storm. There is a considerable degree of ****redundancy (or overlapping activities****) **(** 74) between the (patho)physiological activities of different cytokines, such as TNFα, IL-1, and IL-6, or also between various chemokines. The second characteristics of the cytokine cascade are the ****triggering effects, inducing downstream the expression of many additional cytokines / chemokines together with positive autocrine and paracrine feedback loops as illustrated for TNFα and IL-1 in **Figure 1**. Recently, the amplifying positive feedback loop between IL-6 / STATs and NF-κB signaling has been highlighted with regard to COVID-19 associated mortality (75). Finally, a recent study has shown that the cocktail of the cytokines found most highly upregulated in the circulation of patients with COVID-19 and in PBMCs infected with SARS-CoV-2, *i.e.* IL-6, IL-18, IFN-γ, IL-15, TNF-α, IL-1α, IL-1β, and IL-2 robustly induced cell death the marrow-derived macrophages whereas none of the cytokines individually induced high levels of cell death at the concentration used. Similarly a synergistic effect was found when a cocktail of two cytokines, *i.e.* TNFα and IFNγ, was applied indicating highly synergistic effects between various cytokines on the target cell level (76).

These redundant, triggering, positive feedback-loops (amplifying), and synergistic effects make it utmost difficult to select *the one* crucial cytokine / chemokine within this cascade calling for a systemic approach for simultaneous inhibition of multiple cytokines, including also early expressed cytokines and chemokines.

As summarized in the present review, there is accumulating evidence from recently published studies that indicate the NF-κB signal transduction pathway as a common pathway centrally involved in the generation of the observed cascades of pro-inflammatory cytokines and chemokines in acute respiratory virus infection, including SARS-CoV-2-triggered COVID-19. Reaching beyond the possibilities of currently evaluated drugs for single targets of the cytokine cascade, the inhibition of NF-κB pathway—preferably in parallel at several sensitive points (**Figure 1**)—could provide the unique potential to inhibit the release of multiple cytokines simultaneously, in particular strongly pro-inflammatory cytokines including IL-1, IL-6, TNFα, and chemokines including MIP-1α, MIP-1β, MCP-1, as well as adhesion molecules that are increased during highly inflammatory processes during acute COVID-19 stages.

Multiple approved medications with implicated NF-κB activity involving NSAIDs (*e.g.* acetylsalicylic acid, Aspirin), BTK inhibitors (*e.g.* Ibrutinib, Acalabrutinib), steroids (*e.g.* Dexamethasone), are in wide-spread clinical use and have shown significant clinical efficacy, i.e. significant decrease in mortality was demonstrated (50–52, 54). Although impressive decrease in mortality has been observed, none of these treatments so far has resulted in complete prevention of mortality yet. This might be associated with a still not optimized dosage, start, and duration of treatment. On the other hand, from the sequence of transduction events during NF-kB pathway (see **Figure 1**) it seems plausible that highest effects might be achieved by targeting the latest common steps in NF-κB signal transduction pathway. In this respect inhibiting beside phosphorylation also the following steps of IκB inactivation, *i.e.* ubiquitination and/or proteasome degradation may provide additional treatment options. Several registered proteasome inhibitors (Bortezomib, Carfilzomib, or Ixazomib) are available for treatment of oncological indications (77). This class of substances is known to be powerful inhibitors of NF-κB pathway, with well-known side effect profile known from broad clinical application. In contrast to oncological indications where eight (or more) treatment cycles are routinely applied, it seems plausible that just few applications of proteasome inhibitors will be sufficient to downregulate the acute cytokine storm in COVID-19 patients, with a better side effect profile to be expected (77).

Importantly, in contrast to another recently suggested systemic approach for simultaneous inhibition of cytokines by JAK inhibitors (78), NF-κB inhibition will inhibit predominantly highly pro-inflammatory cytokines and chemokines, such as TNFα, IL-1, IL-6, MCP-1, MIP-1, which are expected to be primarily involved in exuberant systemic inflammatory responses (as proven at the cellular level for COVID-19 patients by the study of Chua et al. (15) rather than cytokines primarily involved in antiviral responsiveness, such as IFNγ (18, 20, 21, 26)—which is primarily dependent on other pathways, i.e. JAK/STAT.

Although there are still many open questions regarding e.g. which compound class—or which combination of—would be most effective, as well as the optimal timing to start treatment (72, 73), the potential to control the cytokine storm-induced severe lung failure and systemic organ failure by using already registered inhibitors of the centrally involved NF-κB pathway may be a real chance to get additional treatment options, hopefully decreasing the number of cases in need for artificial ventilation, multi-organ failure, and death.

## Data Availability Statement

The raw data supporting the conclusions of this article will be made available by the authors, without undue reservation.

## Ethics Statement

The animal study was reviewed and approved by Institutional Animal Care and Use Committee of Tuebingen.

## Author Contributions

RK contributed project idea, discussion of data, writing of manuscript, literature search, and review of manuscript. EH contributed original data. DL contributed original data and discussion of data. WH contributed original data. MO contributed review of manuscript and literature research. OP contributed original data and review of manuscript. All authors contributed to the article and approved the submitted version.

## Conflict of Interest

RK, DL, and WH were employed by Virologik GmbH.

The remaining authors declare that the research was conducted in the absence of any commercial or financial relationships that could be construed as a potential conflict of interest.
